# Acute effect of ambient fine particulate matter on heart rate variability: an updated systematic review and meta-analysis of panel studies

**DOI:** 10.1186/s12199-020-00912-2

**Published:** 2020-12-01

**Authors:** Zhiping Niu, Feifei Liu, Baojing Li, Na Li, Hongmei Yu, Yongbo Wang, Hong Tang, Xiaolu Chen, Yuanan Lu, Zilu Cheng, Suyang Liu, Gongbo Chen, Yuxiao Zhang, Hao Xiang

**Affiliations:** 1 Department of Global Health, School of Health Sciences, Wuhan University, 115# Donghu Road, Wuhan, China; 2 Global Health Institute, Wuhan University, 115# Donghu Road, Wuhan, China; 3 Department of Public Health Sciences, Karolinska Institutet, Tomtebodavägen 18, Solna, SE-171 65 Stockholm, Sweden; 4 School of Management, Chengdu University of Traditional Chinese Medicine, 37# Shierqiao Road, Chengdu, China; 5 Environmental Health Laboratory, Department of Public Health Sciences, University Hawaii at Manoa, Honolulu, HI 96822 USA; 6 School of Chemistry, Chemical Engineering and Life Sciences, Wuhan University of Technology, 122# Luoshi Road, Wuhan, China; 7 Guangdong Provincial Engineering Technology Research Center of Environmental and Health risk Assessment; Department of Occupational and Environmental Health, School of Public Health, Sun Yat-sen University, Guangzhou, China

**Keywords:** Fine particulate matter, Heart rate variability, Panel study, Meta-analysis

## Abstract

**Background:**

Decreased heart rate variability (HRV) is a predictor of autonomic system dysfunction, and is considered as a potential mechanism of increased risk of cardiovascular disease (CVD) induced by exposure to particulate matter less than 2.5 μm in diameter (PM_2.5_). Previous studies have suggested that exposure to PM_2.5_ may lead to decreased HRV levels, but the results remain inconsistent.

**Methods:**

An updated systematic review and meta-analysis of panel studies till November 1, 2019 was conducted to evaluate the acute effect of exposure to ambient PM_2.5_ on HRV. We searched electronic databases (PubMed, Web of Science, and Embase) to identify panel studies reporting the associations between exposure to PM_2.5_ and the four indicators of HRV (standard deviation of all normal-to-normal intervals (SDNN), root mean square of successive differences in adjacent normal-to-normal intervals (rMSSD), high frequency power (HF), and low frequency power (LF)). Random-effects model was used to calculate the pooled effect estimates.

**Results:**

A total of 33 panel studies were included in our meta-analysis, with 16 studies conducted in North America, 12 studies in Asia, and 5 studies in Europe. The pooled results showed a 10 μg/m^3^ increase in PM_2.5_ exposure which was significantly associated with a − 0.92% change in SDNN (95% confidence intervals (95%CI) − 1.26%, − 0.59%), − 1.47% change in rMSSD (95%CI − 2.17%, − 0.77%), − 2.17% change in HF (95%CI − 3.24%, − 1.10%), and − 1.52% change in LF (95%CI − 2.50%, − 0.54%), respectively. Overall, subgroup analysis suggested that short-term exposure to PM_2.5_ was associated with lower HRV levels in Asians, healthy population, and those aged ≥ 40 years.

**Conclusion:**

Short-term exposure to PM_2.5_ was associated with decreased HRV levels. Future studies are warranted to clarity the exact mechanism of exposure to PM_2.5_ on the cardiovascular system through disturbance of autonomic nervous function.

**Supplementary Information:**

The online version contains supplementary material available at 10.1186/s12199-020-00912-2.

## Introduction

Cardiovascular disease (CVD) is the major cause of mortality worldwide, which contributed to 17.8 million deaths in 2017 [1]. In recent years, epidemiologic studies have shown that exposure to particulate matter less than 2.5 μm in diameter (PM_2.5_) increases the risk of CVD [2, 3], and even a short-term exposure to PM_2.5_ may lead to acute cardiovascular events [4–6]. One of the potential mechanisms of PM_2.5_-related acute cardiovascular events is dysfunction of the autonomic nervous system, which is always assessed by the heart rate variability (HRV) levels [7–9]. HRV is regulated by parasympathetic autonomic activation including vagus nerve and sympathetic activation [10], and is usually assessed by time domain indicators (deviation of all normal-to-normal intervals (SDNN), the root mean square of successive differences between normal heartbeats (rMSSD), and frequency domain indicators (high frequency (HF) and low frequency (LF)). The reduction of any of those 4 indicators reflects a dysfunction of the autonomic nervous system [11, 12].

In recent years, many panel-designed studies on the associations between exposure to PM_2.5_ and HRV have been published, which could provide direct evidence for acute health effects of exposure to PM_2.5_ and its potential mechanisms [7, 13]. However, the results remain inconsistent. Some studies reported negative associations between exposure to PM_2.5_ and HRV, whereas others reported no association [5, 14–16]. For example, Wu S et al. examined the relationship between PM_2.5_ exposure and HRV in 11 taxi drivers during the 2008 Olympic Games and found that SDNN and HF change by − 2.2% (95% confidence intervals (95%CI) − 3.8%, − 0.6%) and − 6.2% (95%CI − 10.7%, − 1.5%) with an interquartile range (IQR, 69.5 μg/m^3^) increase of PM_2.5_ exposure, respectively [17]. However, Bartell SM et al. evaluated relationship between exposure to PM_2.5_ and HRV in 50 elderly people with coronary artery disease and did not found significant association in SDNN (percent change = − 0.92%, 95%CI − 3.79%, 1.95%) or rMSSD (percent change = − 0.26%, 95%CI − 4.74%, 4.22%) with an IQR (16.1 μg/m^3^) increase of PM_2.5_ exposure [5]. Wheeler A et al. evaluated the effects of exposure to PM_2.5_ on HRV in 18 chronic obstructive pulmonary disease (COPD) patients and 12 myocardial infarction (MI) patients and observed a significant effect in COPD patients. However, no significant effects were found in MI patients [18]. The inconsistencies in results may be due to different participants, study designs, sample size, PM_2.5_ exposure measurement, and so on.

Meta-analysis study can deal with inconsistent findings to evaluate a pooled effect estimates. Despite 2 meta-analysis studies on the associations between PM_2.5_ exposure and HRV have been published [7, 19], most of included studies were conducted in high-income countries. More than 7 panel-designed studies on the associations between PM_2.5_ exposure and HRV have been published after the latest meta-analysis. Updated studies, especially those conducted in low- and middle-income countries, should be included in the meta-analysis to provide a more comprehensive evidence of effect of PM_2.5_ exposure on HRV. We therefore conducted an updated systematic review and meta-analysis of panel-designed studies to examine the acute health effects of exposure to PM_2.5_ on HRV.

## Methods

### Literature search

Literature was searched in three online databases (PubMed, Embase, and Web of Science), with published date until November 1, 2019. Only panel-designed studies that examined the associations between ambient PM_2.5_ and HRV levels were included. The search strategy was a combination of exposure and outcome including the following three main domains: (1) ambient PM_2.5_ exposure; (2) HRV effects; and (3) panel-designed study [7]. The search strategy was shown in Additional file 1: Appendix A.

We first selected articles by reading titles and abstracts, and then read the full texts of the selected articles to determine whether they should be included in the meta-analysis. Reference lists of all the included studies were also manually searched. Literature was reviewed by two authors independently (ZP N and FF L). Conflicts between the two authors during article selection and data extraction were resolved by discussing with an arbitrator (H X).

### Inclusion and exclusion criteria

Inclusion criteria for articles were (1) original peer-reviewed human subjects research studies, (2) panel-designed studies, (3) published in English, (4) quantitative assessment of outdoor (ambient) PM_2.5_ exposure, and (5) reported to the percent change of HRV with per increment in PM_2.5_ exposure [20]. We excluded studies if they were (1) toxicological studies, summaries or reviews; and (2) focused on indoor or occupational exposure.

### Data extraction

Data were extracted from all eligible studies, including (1) study characteristics: first author, published year, study location, and period; (2) study population: sample size, mean age, the number of males and females, health status; (3) outcome assessment: HRV measures; (4) PM_2.5_ measurement: PM_2.5_ exposure assessment method, exposure-time-window, lag effects, mean and standard deviations (SD) of PM_2.5_ concentrations, increment in PM_2.5_ used in effect estimates; and (5) effect estimates of the association between PM_2.5_ and HRV: percentage change and 95% CIs per 10 μg/m^3^, IQR or SD increment in PM_2.5_ exposure.

Considering that short-term exposure to PM_2.5_ may disturb the autonomic balance for only 1 day [21], and the frequently used exposure-time-window among panel designed studies were within 24 h, we therefore selected results by using PM_2.5_ exposure of the current day and previous one day as the acute HRV effects of exposure to PM_2.5_. For studies provided multiple effect estimates, we selected the representative result based on the following criteria: (1) if a study reported multiple effect estimates of both fixed-sites and individual monitors, we selected the results of individual monitors since individual monitors might reflect the real exposure to PM_2.5_ more accurately; (2) if a study used more than one exposure-time-window, the effect estimate with exposure-time-window nearing 24 h was selected; (3) for study that performed multiple subgroup (locations, health status), we chose the combined effect estimates if it was reported. Otherwise, the results of subgroup were treated as several separate studies; (4) for two or more studies from the same population, only the most recent one with the exposure-time-window nearing 24 h was selected [22]; and (5) for study that performed multiple statistical models, we extracted the results of full-adjusted models.

### Quality assessment

The Newcastle-Ottawa Scale (NOS) was used for quality assessment of included studies. The NOS designed 8 items to assess the critical appraisal of the potential risk of bias. Total score of NOS ranged 0–9. Studies scored higher than or equal to 6 were regarded as high-quality, while those scored less than 6 as low-quality [22]. Two authors (ZP N and FF L) worked independently and inconsistencies in quality assessment were resolved through discussion.

### Statistical analysis

As studies reported effect estimates change in different increments of PM_2.5_ (percent change per 10 μg/m^3^, IQR, or SD increase in PM_2.5_ exposure), we first converted them into a standardized form (percent change per 10 μg/m^3^ increase in PM_2.5_) using the formula as follows:

$$ {\mathrm{Percent}\ \mathrm{change}}_{\left(\mathrm{pre}\ 10\ \upmu \mathrm{g}/{\mathrm{m}}^3\kern0.5em \mathrm{increment}\right)}=\left[{\left(1+{\mathrm{Percent}\ \mathrm{change}}_{\left(\mathrm{original}\right)}\right)}^{\frac{10}{\mathrm{increment}\left(\mathrm{origianl}\right)}}-1\right]\ast 100\% $$

Standard error (SE) for each effect estimate was calculated by using the formula: (Upper limit – Lower limit)/3.92.

Heterogeneity among different studies was examined using Chi-square-based Cochran Q-statistics test and standard *I*^2^. Random-effects model was used to estimate the overall effect. Sources of heterogeneity were explored using subgroup analyses including location (North America, Europe or Asia), exposure assessment method (individual monitor, fixed site, or others), health status of participants (healthy population or patients), and age (< 40 years or ≥ 40 years). The main reason for dividing studies by age of 40 mainly based on the report of World Health Organization (WHO), which defined people aged 40 years or older as high risk of cardiovascular disease [23]. Besides, meta-regression was conducted to explore if heterogeneity was modified by potential modifier (location, PM_2.5_ measurement, health status, age, and PM_2.5_ levels). Subgroup analysis and meta-regression were not performed if the number of one subgroup was less than 5.

The publication bias of included studies was assessed by using funnel plot and Begg’s and Egger’s test. In addition, sensitivity analysis was performed by omitting one study at a time to evaluate if the omission of any study would change the significance of the pooled results. All these statistical analyses were conducted in Stata version 15.0 (StataCorp, College Station, TX, USA).

## Results

### Selection of studies

**Fig. 1:**
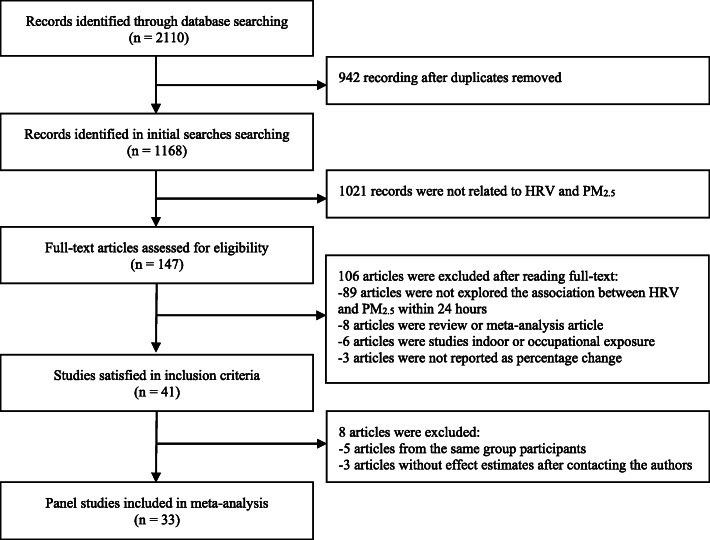
Flow-chart of literature search for meta-analysis

As shown in Fig. 1, 1168 articles were identified in the initial searches after removing duplicates. By reviewing the abstracts, 147 studies were downloaded for full-text reading. Forty-one studies satisfied the inclusion criteria. However, 5 articles were excluded because they were from the same population. Three articles were excluded because there were no effect estimates. Finally, a total of 33 studies were included in our meta-analysis (Fig. 1).

Table 1 provided the characteristics of 33 studies included in meta-analysis. There were 29 studies involving SDNN [5, 14–18, 21, 24–27, 29–31, 35–39, 41–46, 48, 49], 27 studies involving rMSSD [5, 14, 16, 18, 21, 24–26, 29–31, 35–39, 41–43, 45, 46, 49], 24 studies involving HF [5, 14, 16–18, 21, 24–26, 28, 31, 33, 34, 37–39, 42, 43, 49], and 16 studies involving LF [14, 17, 18, 21, 24–26, 31, 34, 37, 38, 42, 43, 49]. The sample size of population participated in panel studies ranged from 9 to 619, and most population were healthy people or patients with CVD. Five studies were conducted in Europe, 16 studies in North America, and 12 studies in Asia. Assessment methods of PM_2.5_ exposure included fixed site (*n* = 17), individual monitors (*n* = 14), and van-based mobile laboratory (*n* = 2) [5, 32]. All included studies adopted linear mixed-effect models to explore associations between exposure to PM_2.5_ and HRV change, and most of them adjusted for temperature, humidity, age, and body mass index (BMI). As for the study quality assessment, all included studies were considered as “high quality” (Table S2).

**Table 1:** Descriptive summaries for all included studies

Reference	Study Location and period	Study population	HRV indices	PM_2.5_ monitoring type, exposure-time-window, lags effects	PM_2.5_ (μg/m^3^)*
Pan L et al. (2018) [24]	Beijing (China); March 28th to May 21th, 2016.	8 elderly subjects with COPD (5 males, 3 female) and 8 Healthy spouses (3 males, 5 female),73 yr.	SDNN, rMSSD, HF, LF	Individual monitor; 12-h average; NA	48.4 ± 45.3
Shutt RH et al. (2017) [25]	Sault Ste (Canada), 5 consecutive 8-h days outdoors in one of two locations (Bayview and College sites). Urban. NA	60 healthy adults (28 males, 32 female), 24.2 yr.	SDNN, rMSSD, LF, HF	Fixed site monitors; 8-h average (averaged over the 6-h period before the visit and 2-h after the visit); NA	College: 11.67 ± 6.61 Bayview: 13.01 ± 6.87
Lim YH et al. (2017) [26]	Seoul (Korea), three health examinations between 2008 and 2010.	466 elderly subjects (111 males, 355 female), 70.6 yr.	SDNN, rMSSD, HF, LF	Fixed monitoring station located on the roof of a building; 24-h average; lag0^#^, lag01^#^, lag02^#^, lag03^#^, lag04^#^, lag05^#^, lag06^#^, lag07^#^, lag08^#^, lag 09 days^#^.	27.0 ± 13.3
Chen SY et al. (2017 )[21]	Taiwan (China), during December 2002 through September 2003.	61 patients with multiple CVD risk factors (33 males, 28 female), 62.9 yr.	SDNN, rMSSD, HF, LF	Fixed air quality monitoring station, Sinjhuang Supersite; 24-h average (1-day lag); lag1 day, lag3 day.	41.4 ± 21.6
Lee MS et al. (2016) [27]	Boston (USA), March 1, 2004, to August 31, 2004.	21 adults without heart disease (4 males, 17 female), 44yr, 19% male.	SDNN	Individual monitor; 5-min average; lag0h-lag4h^#^.	29.8 ± 77.7
Xie Y et al. (2016) [28]	Shanghai (China). September to December 2013.	619 men and women aged from 35–75 yr (235 males, 384 female), 56.7 yr, 38% male.	HF, LF	Individual monitor; 10-h average; NA	90.2 ± 61.3
Peters A et al. (2015) [29]	Germany, March 19, 2007 and December 17, 2008.	64 individuals with type 2 diabetes and impaired glucose tolerance, 66 yr, NA.	SDNN, rMSSD	Central monitoring site; 1-h average; NA	13.7 ± 11.2
Liu WT et al. (2015) [30]	Taiwan (China), between January and March in the years 2012 to 2014.	120 young, healthy subjects (58 males, 62 female), 21.2 yr.	SDNN, rMSSD	Individual monitor; 5-min average; NA	22.3 ± 6.9
Lee MS et al. (2014) [15]	Boston (USA), March to August 2004.	21 community members, 44 yr (4 males, 17 female), NA.	SDNN	Individual monitoring; 5-min average; NA.	29.8 ± 77.7
Xu MM et al. (2013) [31]	Beijing (China), July 2007–September, 2008.	42 patients with heart disease (14 males, 28 female), 54–78 yr.	SDNN, rMSSD, HF, LF	Fixed site monitors; 20-h average; lag0, lag1 (21–40 h), lag2 (41–60 h).	80.6 ± 35.0
Bartell SM et al. (2013) [5]	Los Angeles (USA), 2005 to 2007.	50 participants from four retirement communities with coronary artery disease (31 males, 19 female), ≥ 71 yr.	SDNN, rMSSD	Individual monitor; 1 h^#^, 4 h^#^, 8 h^#^, 24 h average, 3 days^#^, 5 days^#^; NA.	21.1 ± 11.4
Shields KN et al. (2013) [32]	Mexico City Metropolitan Area (Mexico), February 11 to 23, 2002.	16 researchers (11 males, 5 female), 22–56 yr,.	SDNN, HF, LF	Van-based mobile laboratory: 5-min average, 30-, 60-, and 90-min; NA.	14 ± 8
Huang J et al. (2013) [33]	Beijing (China), May 2011 to October 2011.	40 young healthy adults (16 males, 24 female), 24.4 yr.	SDNN, rMSSD, HF, LF	Individual monitor; 5-min average, 15 min^#^, 30 min^#^, 1 h^#^; NA.	Median: Traffic center:162.10 Park:53.00
Jia X et al. (2012) [34]	Beijing (China), August 2008 to September 2008.	30 healthy elderly subjects (12 males, 18 female), 57.9 yr.	HF, LF	Fixed monitors on a six-floor rooftop; 5-min average, 15 min^#^ 30 min^#^, 1 h^#^, 2 h^#^, 4 h^#^ ,6 h^#^; NA.	median: 44.09
Rich DQ et al. (2012) [35]	New York state (USA), June 2006 to November, 2009.	76 participants with MI or unstable angina (51 males, 25 female), NA, 67% male.	SDNN, rMSSD	Fixed site monitors: a wide range particle spectrometer; 31-h average (averaged over the 24-h period before the visit as well as a shorter lag period (lag 0–5 h)); lag h 24–47 h^#^, 48–71 h^#^, 72–95 h^#^, 96–119 h^#^.	8.67 ± 6.06
Hampel R et al. (2012) [36]	Augsburg (Germany), March 2007 to December, 2008.	61 subjects with diabetes or impaired glucose tolerance (40 males, 21 female), 67.5 yr.	SDNN, rMSSD	Fixed site monitors in Augsburg; 1-h averages; lag1h, lag2h lag3h, lag4h, lag 5h, lag 6h.	13.7 ± 11.2
Huang W et al. (2012) [37]	Beijing (China), during summer 2007 and summer 2008.	40 subjects with CVD (16 males, 24 female), 65.6 yr.	SDNN, rMSSD, HF, LF	Air monitoring station; 1 h^#^, 4 h average, 12 h^#^; NA	Visit 1: 112.5 ± 61.3 Visit 2: 78.3 ± 50.6 Visit 3: 89.2 ± 53.9 Visit 4: 64.2 ± 39.9
Wu CF et al. (2010) [14]	Taiwan (China), February to March 2007.	17 mail carriers (17 males, 0 female), 32.4 yr, 100% male.	SDNN, rMSSD, HF, LF	Individual monitor; 7-h average; NA.	68.2 ± 30.0
Wu S et al. (2010) [17]	Beijing (China), May 2008-March 2009.	9 healthy taxi drivers (4 males, 5 female), 27–39 yr.	SDNN, rMSSD, HF, LF	Individual monitor;30-mim average, 2-h average; NA	Before: 95.4 ± 58.6; During: 39.5 ± 25.2; After: 64.0 ± 80.3
Chuang KJ et al. (2007) [38]	Taiwan (China), April-June of 2004 or 2005.	76 healthy college students (46 males, 30 female), 21 yr.	SDNN, rMSSD, HF, LF	Fixed site: 1-day average, 2-day, 3-day; NA.	31.8 ± 10.6
Zanobetti A et al. (2010) [39]	Boston (USA), 1999 to 2003.	46 patients with coronary artery disease (37 males, 19 female), 43–75 yr.	SDNN, rMSSD, HF	Fixed site monitors: 30 min^#^, 1 h^#^, 2 h average, 48 h^#^, 72 h^#^, 96 h^#^ ,120 h^#^; NA.	Median: 9.54
Suh HH et al. (2010) [16]	Atlanta (USA), Fall 1999 and Spring 2000.	30 subjects: 12 with a recent MI and 18 with COPD (17 males, 13 female), 65 yr.	SDNN, rMSSD, HF, LF	Individual monitor; 24-h Ambient, 24h personal; NA.	15.78 ± 8.75
Schneider A et al. (2010) [40]	Erfurt (Germany), October 2000 and April 2001.	56 patients with ischemic heart disease, stable angina pectoris or prior myocardial infarction at an age of at least 50 years (56 males, 0 female). 66 yr.	SDNN, rMSSD, HF, LF	Fixed monitoring site: 24-h average; NA	20.3 ± 14.8
Folino AF et al. (2009 )[41]	Padua (Italy), Summer 2006, Winter and Spring 2007.	39 patients with MI (36 males, 3 female),45–64 yr, 92% male	SDNN, rMSSD	Individual monitor: 24-h average; NA	Visit 1: 33.9 ± 12.7; Visit 2: 62.1 ± 27.9; Visit 3: 30.8 ± 14.0
Yeatts K et al. (2007) [42]	North Carolina (USA), a 12-week period, September 2003 to July 2004.	12 adult asthmatics (3 males, 8 female), 33 yr,.	SDNN, rMSSD	Fixed site monitors:24-h average; NA	12.5 ± 6.0
Adar SD et al. (2007) [43]	Missouri (USA), March and June of 2002.	44 nonsmoking seniors (7 males, 37 female), 62–94 yr, 16% male.	SDNN, rMSSD, HF, LF	Individual monitor: 5-min^#^, 30-min^#^, 1-h^#^, 4-h^#^, and 24-h average; NA.	7.7 ± 6.8
Luttmann-Gibson H et al. (2006) 40]	Ohio (USA), during summer (June 4 to August 18) and fall (September 25 to December 15) of 2000.	32 nonsmoking senior adults (3 males, 19 female), 70.8 yr, 9% male.	SDNN, rMSSD, HF, LF	Ambient monitoring site at located at the Franciscan University: 24-h average; NA.	19.7
Wheeler A et al. (2006) [18]	Atlanta (USA), In fall 1999 and spring 2000.	18 individuals with COPD and 12 individuals with MI (17 males, 13 female), 65 yr, 57% male.	SDNN, rMSSD, HF, LF	Fixed site monitors; 4-h average; NA.	17.8
Timonen KL et al. (2006) [44]	Erfurt (Germany), Helsinki (Finland), Amsterdam (Netherlands),1998 to 1999.	131 subjects with stable coronary artery disease (90 males, 41 female), 40–84 yr.	SDNN, HF	Fixed site monitors; 24-h average**,** 5-day average**;** lag0, lag1, lag2, lag3 day.	Amsterdam: 20.0; Erfurt: 23.1; Helsinki:12.7
Riediker M et al. (2004) [45]	North Carolina (USA), Fall 2001.	9 healthy policemen (9 males, 0 female),23–30 yr.	SDNN, HF, LF	Individual monitor; 24-h average; NA.	23
Schwartz J et al. (2005) [46]	Massachusetts (USA), 12 weeks during the summer of 1999.	28 elderly subjects aged 61–89 yr (7 males, 14 female), 71 yr.	SDNN, rMSSD	Fixed monitoring site at Harvard University: 1-h average; NA.	Median: 10
Magari SR et al. (2002) [47]	USA, June to December 1999.	20 relatively young, healthy male workers (20 males, 0 female), 43 yr, 100% male.	SDNN	Individual monitoring: 3-h average; NA	96 ± 158
Brauer M et al. (2001) [48]	Vancouver (Canada), April-September. 1998.	16 patients with COPD (7 males, 9 female), 54–86 yr.	SDNN, rMSSD	Individual monitor; 24-h average; NA.	18.2 ±14.6

^#^ Studies performed more than one exposure-time-window or lag effects; however, the results were just presented as figures and the effect estimates were not reported. Linear mixed-effects models were all used statistical approach; HRV measures were all log-transformed; PM_2.5_ levels were showed by the mean ± standard deviation (SD) or mean if it was not specified. *PM*_*2*.5_ particulate matter with aerodynamic diameter equal to or less than 2.5 μm, *HRV* heart rate variability, *COPD* chronic obstructive pulmonary disease, *CVD* cardiovascular disease, *MI* prior myocardial infarction, *SDNN* standard deviation of all normal-to-normal intervals, *rMSSD* root mean square of successive differences in adjacent normal-to-normal intervals, *HF* high-frequency power, *LF* low-frequency power, *IHD* ischemic heart disease, *LF* low-frequency band (0.04 to 0.15 Hz), *yr* year, *h* hour, *NA* not reported. Visit 1 represented the first follow-up during the study period, visit 2 represented the second follow-up during the study period. etc.; Before, During, and After represented the clinical visits before, during and after the Beijing Olympic Games

### Time-domain measures of HRV and PM_2.5_ exposure

**Fig. 2:**
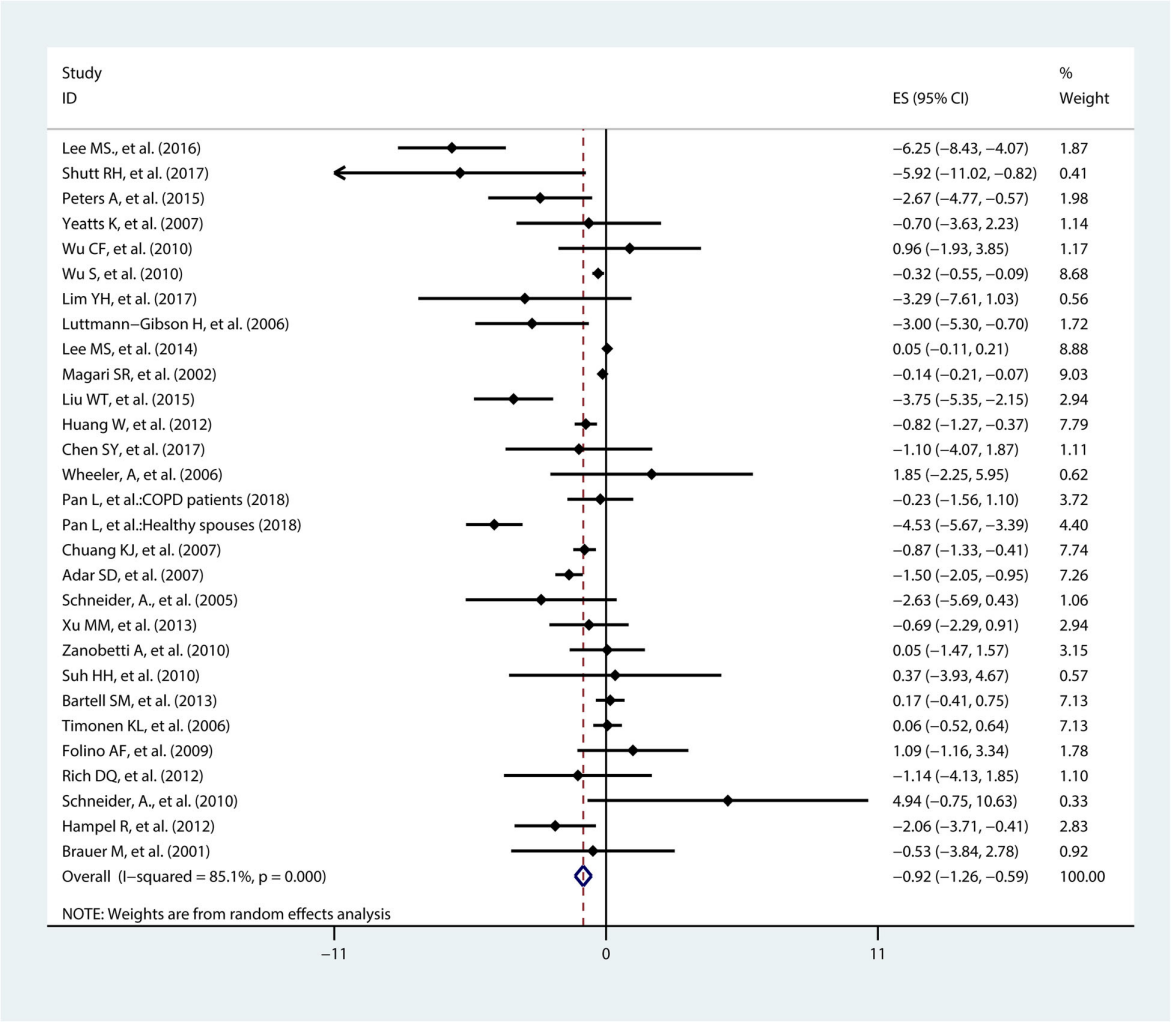
Pooled percent change (%) of SDNN associated with a 10 μg/m^3^ increase of PM_2.5_

The pooled effected estimates from 29 studies on SDNN showed a 10 μg/m^3^ increase in exposure to PM_2.5_ was associated with a − 0.92% change in SDNN (95%CI − 1.26%, − 0.59%) (Fig. 2). Subgroup analysis by location suggested a larger variation of SDNN in Asian (percent change = − 1.38%, 95%CI − 2.13%, − 0.62%) than in European (percent change = − 0.85%, 95%CI − 2.39%, 0.70%) and North American populations (percent change = − 0.62%, 95%CI − 1.05%, − 0.19%) after short-term exposure to PM_2.5_. Subgroup analysis by health status indicated that a 10 μg/m^3^ increase in exposure to PM_2.5_ was associated with a − 0.67% change (95%CI − 1.02%, − 0.32%) in SDNN among healthy population, which was smaller than in patients (percent change = − 1.19%, 95%CI − 2.04%, − 0.34%). In addition, subgroup analysis by age indicated that the decrease level of SDNN caused by PM_2.5_ exposure among people aged over 40 (percent change = − 0.97%, 95%CI − 1.37%, − 0.58%) was similar with those aged under 40 years (percent change = − 0.87%, 95%CI − 1.77%, 0.03%) (Table 2).

**Table 2:** Summary of percent change estimates (95%CI) for included studies of association between PM_2.5_ exposure (per 10 μg/m3 increase) and HRV indicators

Subgroup	SDNN				rMSSD				HF				LF			
	No.	Percent Change (95%CI)	*I*^2^ (%)	*P*	No.	Percent Change (95%CI)	*I*^2^ (%)	*P*	No.	Percent Change (95%CI)	*I*^2^ (%)	*P*	No.	Percent Change (95%CI)	*I*^2^ (%)	*P*
Total	29	− 0.92 (− 1.26, − 0.59)	85.1	< 0.001	27	− 1.47 (− 2.17, − 0.77)	79.3	< 0.001	24	− 2.17 (− 3.24, − 1.10)	88.9	< 0.001	16	− 1.52 (− 2.50, − 0.54)	72.4	< 0.001
Study location																
North American	15	− 0.62 (− 1.05, − 0.19)	82.0	< 0.001	13	− 1.64 (− 2.89, − 0.38)	79.8	< 0.001	10	− 1.18 (− 3.22, 0.86)	77.4	< 0.001	6	− 1.39 (− 2.99, 0.20)	54.2	0.053
Europe	4	− 0.85 (− 2.39, 0.70)	75.3	0.007	5	− 0.90 (− 2.47, 0.67)	80.9	< 0.001	2	NA	−	−	0	NA	NA	NA
Asia	10	− 1.38 (− 2.13, − 0.62)	87.2	< 0.001	9	− 1.58 (− 2.43, − 0.73)	62.2	< 0.001	12	− 2.54 (− 3.91, − 1.17)	91.8	< 0.001	10	− 1.63 (− 2.9, − 0.32)	78.7	< 0.001
PM_2.5_ assessment																
Individual monitor	14	− 0.98 (− 1.40, − 0.55)	91.3	< 0.001	10	− 1.54 (− 2.55, 0.53)	84.1	< 0.001	11	− 3.84 (− 5.03, − 1.92)	93.0	< 0.001	6	− 1.30 (− 2.79, 0.20)	84.1	< 0.001
Fixed site or others	15	− 0.94 (− 1.56, − 0.32)	56.7	0.004	17	− 1.36 (− 2.30, − 0.42)	68.7	< 0.001	13	− 0.73 (− 2.14, 0.67)	68.9	< 0.001	10	− 1.75 (− 3.14, − 0.37)	54.7	0.019
Health status																
Healthy population	13	− 0.67 (− 1.02, − 0.32)	84.7	< 0.001	10	− 2.18 (− 3.39, − 0.97)	71.7	< 0.001	11	− 3.40 (− 4.97, − 1.83)	93.8	< 0.001	8	− 1.91 (− 3.25, − 0.57)	80.6	< 0.001
Patients	16	− 1.19 (− 2.04, − 0.34)	84.1	< 0.001	17	− 1.08 (− 1.85, − 0.31)	74.3	< 0.001	13	− 0.90 (− 2.34, 0.54)	71.4	< 0.001	8	− 1.03 (− 2.73, 0.68)	61.2	< 0.001
Age																
<40 years	6	− 0.87 (− 1.77, 0.03)	80.0	< 0.001	5	− 0.20 (− 2.64, 2.23)	79.8	< 0.001	6	− 1.19 (− 2.53, 0.15)	84.7	< 0.001	5	− 0.71 (− 1.32, − 0.11)	75.8	< 0.001
≥ 40 years	23	− 0.97 (− 1.37, − 0.58)	85.8	< 0.001	22	− 1.64 (− 2.39, − 0.88)	81.	< 0.001	18	− 2.70 (− 4.35, − 1.05)	91.9	< 0.001	11	− 2.23 (− 4.00, − 0.46)	17.4	< 0.001

P for the heterogeneity *Q* test. *NA* not reported, due to the limited number of studies in this subgroup, the subgroup analysis was not conducted

*HRV* heart rate variability, *SDNN* standard deviation of all normal-to-normal intervals, *rMSSD* root mean square of successive differences in adjacent normal-to-normal intervals, *HF* high frequency power, *LF* low frequency power

**Fig. 3:**
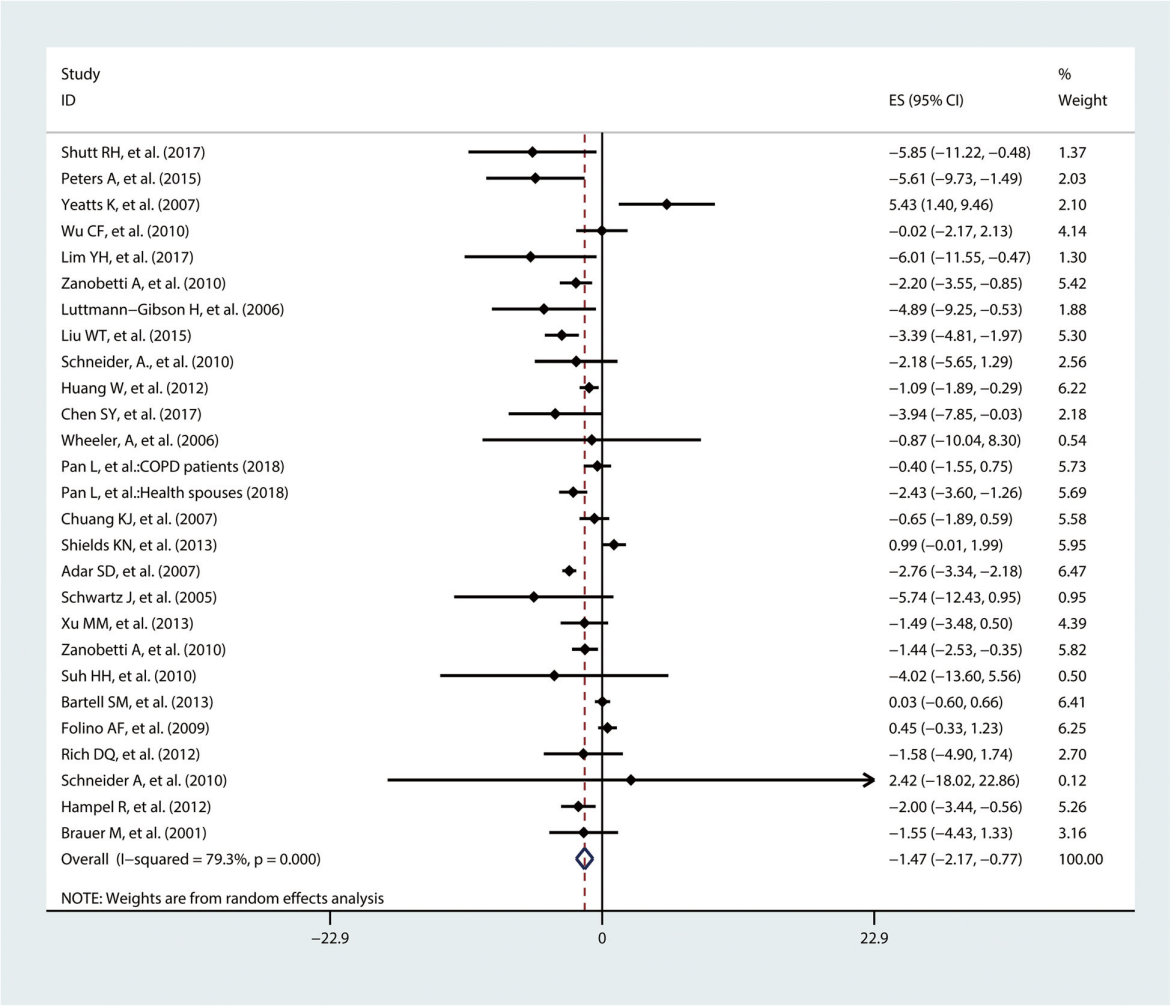
Pooled percent change (%) of rMSSD associated with a 10 μg/m^3^ increase of PM_2.5_

In total, 27 studies investigated the association of short-term exposure to PM_2.5_ with rMSSD. Meta-analysis showed that a 10 μg/m^3^ increase in exposure to PM_2.5_ was associated with a − 1.47% change in rMSSD (95%CI − 2.17%, − 0.77%) (Fig. 3). Subgroup analysis by study location suggested that the adverse effect of PM_2.5_ on rMSSD was similar among North American populations (percent change = − 1.64%, 95%CI − 2.89%, − 0.38%) and Asian populations (percent change = − 1.58%, 95%CI − 2.43%, − 0.73%). Short-term exposure to PM_2.5_ was also associated with decreased rMSSD levels in Europeans, although the association was not statistically significant (percent change = − 0.90%, 95%CI − 2.47%, 0.67%). Furthermore, subgroup analyses by health status showed that the decrease level of rMSSD caused by PM_2.5_ among healthy population (percent change = − 2.43%, 95%CI − 3.40%, − 1.45%) was larger than in patients (percent change = − 0.87%, 95% CI − 1.58%, − 0.77%). Subgroup analysis by age suggested that the decrease level of rMSSD caused by PM_2.5_ among people aged over 40 (percent change = − 1.64%, 95%CI − 2.39%, − 0.88%) was larger than those aged under 40 (percent change = − 0.20%, 95%CI − 2.64%, 2.23%) (Table 2).

### Frequency-domain indices of HRV and PM_2.5_ exposure

**Fig. 4:**
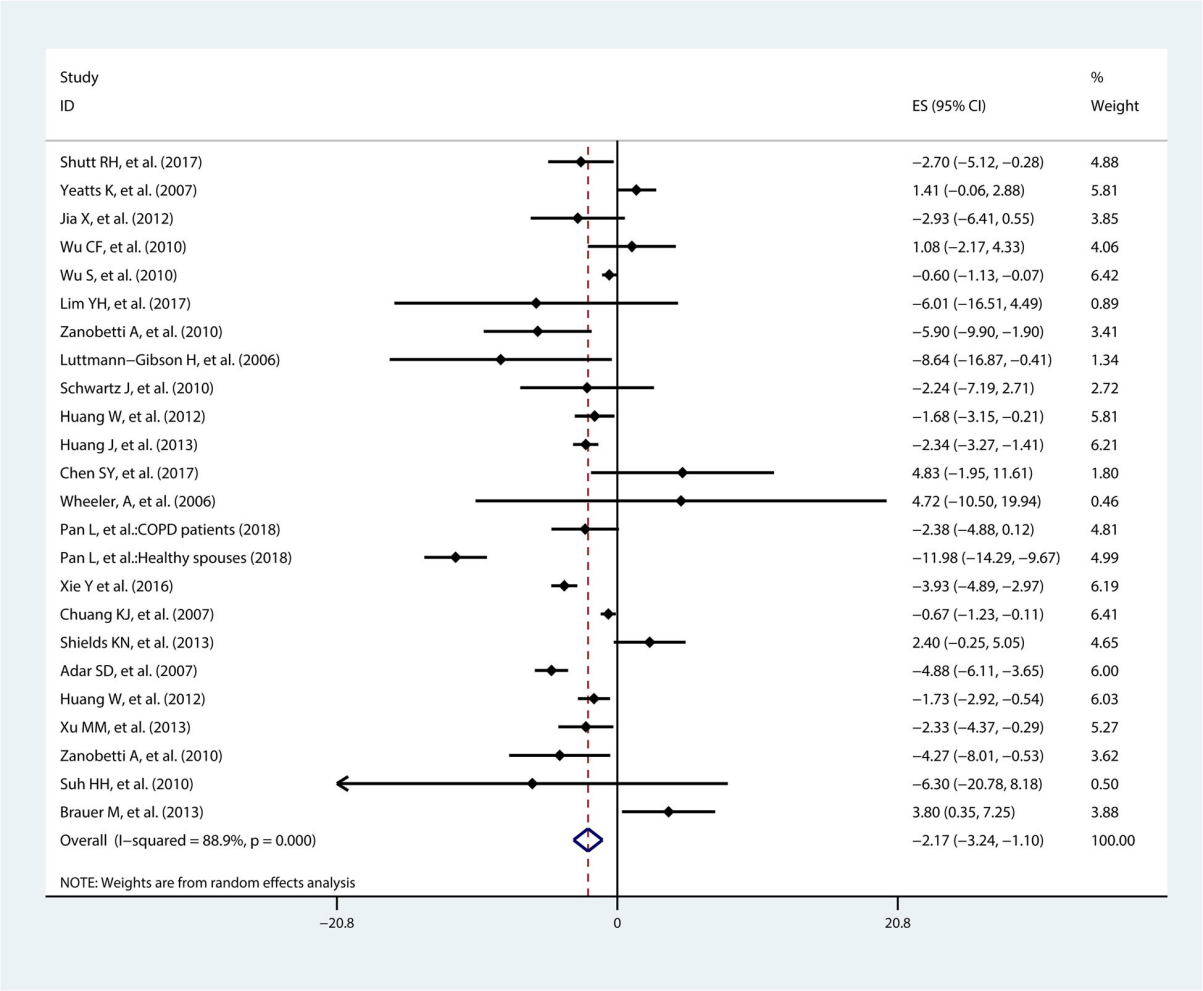
Pooled percent change (%) of HF associated with a 10 μg/m^3^ increase of PM_2.5_

The pooled effect estimates from 24 studies on HF suggested a negative effect. HF changed by − 2.17% (95%CI − 3.24%, − 1.10%) when PM_2.5_ concentration increased per 10 μg/m^3^ (Fig. 4). Subgroup analyses by location showed that decrease of HF level in Asia (percent change = − 2,54%, 95%CI − 3.91%, − 1.17%) was larger than in North American populations (percent change = − 1.18%, 95%CI − 3.22%, 0.86%). Subgroup analyses by health status showed that the decrease of HF in patient was − 0.90% (95%CI − 2.34%, 0.54%), which was smaller than decrease of HF in healthy population (percent change = − 3.40%, 95%CI − 4.97%, − 1.83%). Moreover, subgroup analysis by age suggested a greater decreased HF in people aged over 40 years (percent change = − 2.70%, 95%CI − 4.35%, − 1.05%) than those aged under 40 years (percent change = − 1.19%, 95%CI − 2.35%, 0.15%). Subgroup analyses by PM_2.5_ assessment did not show a remarkable difference (Table 2).

**Fig. 5:**
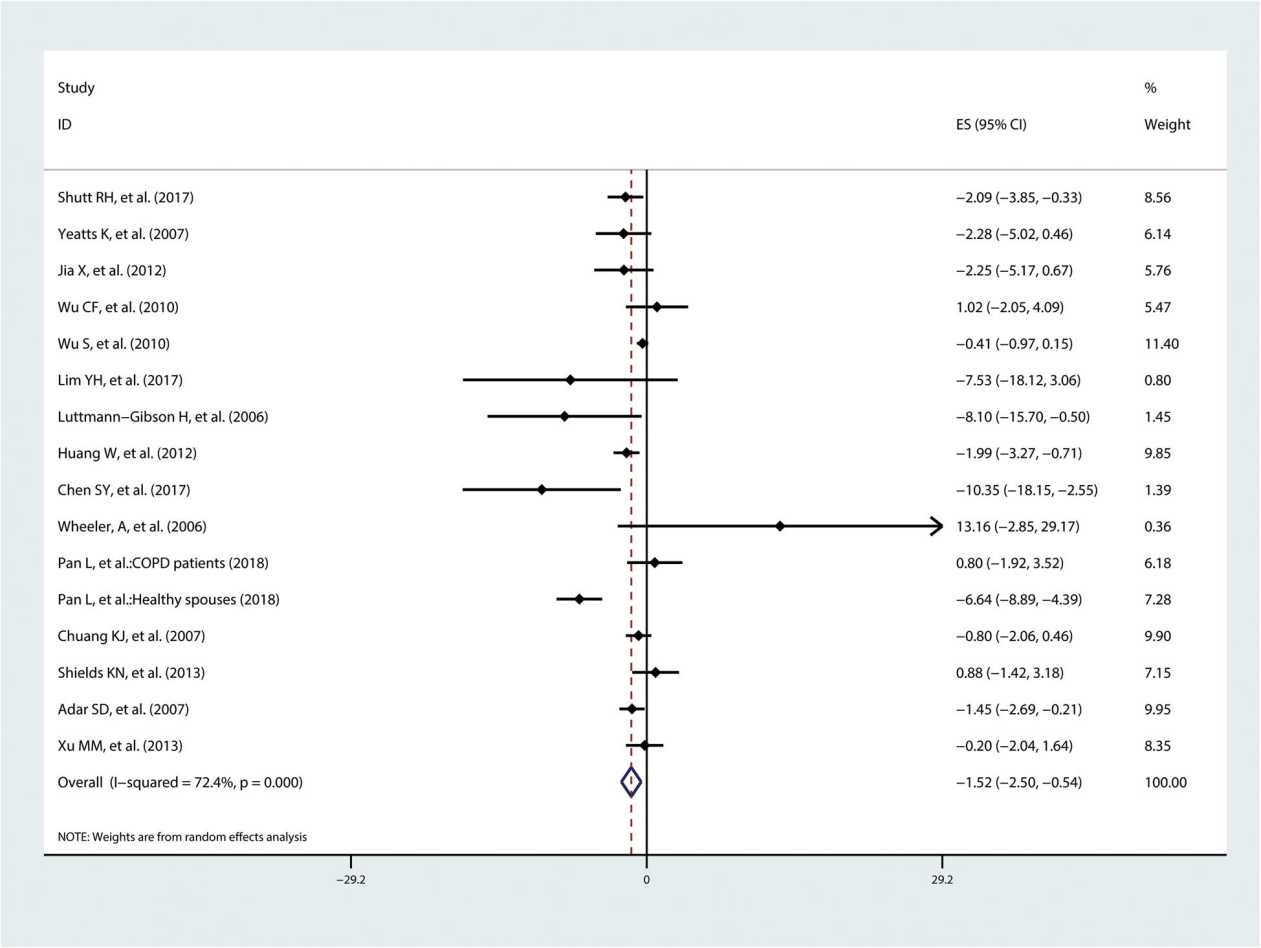
Pooled percent change (%) of LF associated with a 10 μg/m^3^ increase to PM_2.5_

A total of 16 studies investigated the associations between exposure to PM_2.5_ and LF. Meta-analysis suggested that LF would change by − 1.52% (95%CI − 2.50%, − 0.54%) with a 10 μg/m^3^ increment of PM_2.5_ (Fig. 5). Subgroup analyses by location suggested a − 1.64% (95%CI − 2.94%, − 0.32%) change of LF level in Asia, while a weak but not statistically significant association in North American populations (percent change = − 1.39%, 95%CI − 2.99%, 0.20%). Subgroup analyses by health status revealed a greater effect in healthy population (percent change = − 1.91%, 95%CI − 3.25%, − 0.57%) than patients (percent change = − 1.03%, 95%CI − 2.73%, 0.68%). Besides, subgroup analyses by age showed a higher effect estimate in people aged over 40 years (percent change = − 2.23%, 95%CI − 4.00%, − 0.46%) than people aged under 40 years (percent change = − 0.71%, 95%CI − 1.32%, − 0.11%) (Table 2).

### Heterogeneity, meta-regression analysis, publication bias, and sensitivity analysis

Heterogeneity existed in all four measures of HRV (*I*^2^ > 50%, *P* < 0.001). The meta-regression analysis identified that health status could explain the heterogeneity for rMSSD change associated with exposure of PM_2.5_ (Table S4, Figure S1, Figure S2). Funnel plots of PM_2.5_ and rMSSD, HF and LF showed a slight asymmetry, but the *P* values of Begg's test and Egger’s test were all greater than 0.05, demonstrating that publication bias were acceptable (Table S3, Figure S3). However, publication bias may exist among studies on SDNN, since the *P* value of Egger’s test was less than 0.05. In sensitivity analyses, we found that the average percent changes in time (SDNN, rMSSD) and frequency domains (LF, HF) of HRV were all in the combined confidence interval, suggesting that the results of the meta-analysis were reliable and stable (Figure S4).

## Discussion

Our meta-analysis demonstrated that exposure to ambient PM_2.5_ was significantly associated with decreased HRV levels, suggesting that PM_2.5_ may increase the risk of CVD through automatic nervous system dysfunction. A 10 μg/m^3^ increase in PM_2.5_ exposure was associated with a − 0.92% change in SDNN, − 1.47% change in rMSSD, − 2.17% change in HF, and − 1.52% change in LF, respectively.

Previous meta-analyses indicated that increased exposure to PM_2.5_ was negatively associated with HRV levels [7, 19]. For example, Pieters N et al. reported that a 10 μg/m^3^ increase in PM_2.5_ was associated with a − 0.12% change in SDNN (95%CI − 0.22%, − 0.03%), − 2.18% change in rMSSD (95%CI − 3.33%, 1.03%), and − 2.44% change in HF (95% CI − 3.76%, − 1.12%), respectively [19]. Buteau S et al. reported that a 10 μg/m^3^ increase in PM_2.5_ was associated with a − 2.11% change in SDNN (95%CI − 4.00%, − 0.23%), − 3.29% change in rMSSD (95%CI − 6.32% , − 0.25%), − 4.76% change in LF (95%CI − 12.10%, 2.58%), and − 1.74% change in HF (95%CI − 7.79%, 4.31%), respectively [7]. The pooled effects of Buteau’ study was much higher than our study, especially for rMSSD and LF, possibly because Buteau’ study only conducted the meta-analysis among the older participants.

The results of subgroup analysis showed that percent changes of HRV for a 10 μg/m^3^ PM_2.5_ increase among Asians were larger than in North American populations and European populations, which may be attributed to the serious environmental pollution and different composition of particulate matters [50, 51]. Previous studies have reported the air pollution levels in many Asian countries, such as China, were 10 times higher than that in Europe and North America [52]. Combined with the results of our study that the effect estimates of exposure to PM_2.5_ on HRV in Asia were higher than that in Europe and Northern America, it may partly explain why the risk of PM_2.5_ on cardiovascular diseases/mortality is higher in Asian countries [53, 54].

We found that the decreased levels of rMSSD, HF, and LF were greater in healthy population than in patients, since patients may take anti-autonomic nerve dysfunction medication, such as benazepril, nimodipine, and thus weaken the effect of PM_2.5_ on HRV response. In addition, subgroup analysis by age in our study showed a higher effect estimates in people aged over 40 compared with those aged under 40. Previous studies also reported that elders were more vulnerable to the PM_2.5_-related risk of decreased HRV levels and adverse cardiovascular events [55–57]. For example, the European Study of Cohorts for Air Pollution Effects (ESCAPE) project reported that participants aged over 60 years were more sensitive to PM_2.5_ exposure than the younger participants [58].

Potential pathophysiological mechanisms included autonomic imbalance, increased oxidative stress, and inflammation, through which PM_2.5_ may accelerate the development of CVD [59]. The dysfunction of the autonomic nervous system has been found as the major pathway that result in PM_2.5_-related adverse cardiovascular outcomes [7–9]. Both toxicology experiments and epidemiological studies have provided substantial evidence that PM_2.5_ exposure would decrease HRV level, and then lead to autonomic nervous dysfunction, which subsequently increase the risk of CVD [21, 24–26, 60]. For example, Chiarella SE et al. found that the levels of alveolar lavage fluid and plasma catecholamine in mice increased after inhaling PM_2.5_, with the activation of sympathetic nervous system [60]. Chen SY et al. examined the effects of short-term exposue to urban air pollution among 61 high-risk CVD subjects and found that PM_2.5_ caused an immediate autonomic nervous dysfunction as well as long-term inflammatory and thrombotic responses [21].

Some limitations of our meta-analysis should be noticed. Firstly, subgroup analysis and meta-regression by location, PM_2.5_ measurement, and age group did not explain the observed heterogeneity. Meta-regression suggested that heterogeneity among exposure to PM_2.5_ and rMSSD changes may be explained by health status. Secondly, we could not assess the effect of lag more than 24 h of PM_2.5_ exposure, because there were large differences on the reported lag effects of PM_2.5_ exposure among different studies and many studies were not represented in the percent change of lag effects. Finally, we failed to explore gender disparities in associations between PM_2.5_ exposure and HRV levels because most of the panel studies including our meta-analysis only reported the combined effects estimates both male and female participants and subgroup effects estimates were not represented.

## Conclusion

Our study demonstrated that exposure to PM_2.5_ was associated with decreased levels of HRV, suggesting that exposure to ambient PM_2.5_ may increase CVD risk through the activation of autonomic nervous system. Further studies should be conducted to clarity the specific mechanism of exposure to PM_2.5_ on health effects.

## Supplementary Information

**Additional file 1.**

## Data Availability

All the data generated or analyzed during this study are included in this published article.
