# Supplementary material for: Acute effect of ambient fine particulate matter on heart rate variability: an updated systematic review and meta-analysis of panel studies
Source: Environ Health Prev Med. 2020 Dec 1;25:77. doi: 10.1186/s12199-020-00912-2 (PMC7706193; doi:10.1186/s12199-020-00912-2)
Supplement: Supplementary file 1 — Additional file 1. [file 12199_2020_912_MOESM1_ESM.docx]

**Supplementary Materials**

**Acute effect of ambient fine particulate matter on heart rate variability: an updated systematic review and meta-analysis of panel studies**

Zhiping Niu^1,2^ ^*^, Feifei Liu ^1,2^ ^*^, Baojing Li^3^, Na Li^1,2^, Hongmei Yu^4^, Yongbo Wang^1^, Hong Tang^1,2^, Xiaolu Chen^1,2^, Yuanan Lu^5^, Zilu Cheng ^6^, Suyang Liu^1,2^, Gongbo Chen^7^, Yuxiao Zhang ^1,2 †^, Hao Xiang^1,2 †^

Appendix A - Search Strategy.

Appendix B. The derivation process of the formula applied in our study.

Appendix C - Table S1. PRISMA report checklist.

Appendix D - Table S2. Quality assessment of included studies.

Appendix E - Table S3. Publication bias.

Appendix F - Table S4: Meta-regression analysis by potential modifier.

Appendix G - Figure S1. Meta-regression plots of percent change of HRV and age of participants.

Appendix H- Figure S2. Meta-regression plots of percent change of HRV and PM_2.5_ levels.

Appendix I - Figure S3. Funnel plots of publication bias analyses.

Appendix J- Figure S4. Sensitivity analysis the lower and upper limits of the pooled results after omitting one study each at a time.

**Appendix A - Search Strategy**

PubMed
Concept 1: Ambient air pollution

#1 Air pollutants [Title/Abstract] OR Air pollution[Title/Abstract] OR Air pollutant[Title/Abstract] OR Particulate matter[Title/Abstract]) OR air pollut*[Title/Abstract] OR particle*[Title/Abstract] OR particulate matter*[Title/Abstract] OR particulate air pollutant*[Title/Abstract]

Concept 2: Cardiovascular effects

#2 Heart rate [MeSH Terms] OR Inflammation [MeSH Terms] OR Autonomic nervous system [MeSH Terms] OR Blood Physiological Phenomena [MeSH Terms] OR Cardiovascular Physiological Processes [MeSH Terms] OR Cardiovascular Physiological Phenomena [MeSH Terms]

#3 Heart rate variability OR cardiovascular inflammation OR cardiovascular function OR
autonomic nervous system [All Fields]

#4 (cardiovascular OR cardio-vascular OR cardiac OR heart OR vascular) AND (effect*[Abstract] OR rate* OR change* OR impact* OR dysfunction*)[All Fields]
#5 (#2 OR #3 OR #4)

Concept 3: Longitudinal (panel) study
#6 Longitudinal studies OR Longitudinal study OR Panel study OR Prospective study OR Follow up OR (follow-up or followed* or repeat* or panel* or longitudinal or cohort* or prospective) [All Fields] #7 English [Language]
#8 (#1 AND #5 AND #6 AND #7)

Embase

Concept 1: Ambient air pollution
#1 (air pollutants OR air pollution OR air pollutant OR particulate matter OR air pollut* OR
particle* OR particulate matter* OR particulate air pollutant) ti,ab,kw

Concept 2: Cardiovascular effects
#2 (Heart rate OR Inflammation OR Autonomic nervous system OR Cardiovascular Physiological Processes OR 'cardiovascular function' OR heart rate variability OR cardiovascular inflammation OR 'cardiovascular function') exp
#3 heart rate OR inflammation OR autonomic nervous system disease OR cardiovascular physiological processes OR cardiovascular physiological phenomena OR heart rate variability OR cardiovascular inflammation OR cardiovascular function
#4 (#2 OR #3)

Concept 3: Longitudinal (panel) study
#5 Longitudinal studies OR Longitudinal study OR Panel study OR Prospective study OR Follow up OR (follow-up OR followed* OR repeat* OR panel* OR longitudinal OR cohort* or 'prospective study') #6 English [Language]

#7 #1 AND #4 AND #5 AND #6

**Web of Science**
**Concept 1: Ambient air pollution**
#1 TS= (air pollution OR air pollutant OR pollution OR pollutant* OR particulate matter OR particulate matters OR particulate* OR particle*)
**Concept 2: Cardiovascular effects**
#2 TS= (Heart rate OR Inflammation OR Autonomic nervous system disease OR Blood Physiological Phenomena OR Cardiovascular Physiological Processes OR Cardiovascular Physiological Phenomena OR Heart rate variability OR cardiovascular inflammation OR cardiovascular function)
**Concept 3: Longitudinal (panel) study**
#3 TS= (Longitudinal studies OR Longitudinal study OR Panel study OR Prospective study) #4 language= English
**#5 #1AND #2 AND #3 AND #4**

**Appendix B. The derivation process of the formula applied in our study.**

$${Percent change}_{\left( \Delta1 \right)}=[\left( 10 \right)^{\beta*\Delta1}-1]*100\% （1）$$

$$\beta*\Delta1=lg(1+{Percent change}_{\left( \Delta1 \right)}) （2）$$

$$\beta*\Delta1*\frac{\Delta2}{\Delta1}=\frac{\Delta2}{\Delta1}*lg(1+{Percent change}_{\left( \Delta1 \right)}) （3）$$

$$\beta*\Delta2=lg\left( 1+{Percent change}_{\left( \Delta1 \right)} \right)^{\frac{\Delta2}{\Delta1}} （4）$$

$$\left( 10 \right)^{\beta*\Delta2}=\left( 1+{Percent change}_{\left( \Delta1 \right)} \right)^{\frac{\Delta2}{\Delta1}} （5）$$

$${Percent change}_{\left( \Delta2 \right)}=\left[ \left( 10 \right)^{\beta*\Delta2}-1 \right]*100\% =\left[ \left( 1+{Percent change}_{\left( \Delta1 \right)} \right)^{\frac{\Delta2}{\Delta1}}-1 \right]*100\% （6）$$

Formula from (3) to (4) is: n_*_log_a_ M= log_a_ M^n^. ∆_1_, ∆_2_ was increment of PM_2.5_; Percent change _(∆1)_ was the percentage change of HRV when PM_2.5_ increased ∆_1_ μg/m^3^, Percent change _(∆2)_ was the percentage change of HRV when PM_2.5_ increased ∆_2_ μg/m^3^; β was regression coefficient of linear mixed effects models.

| **Appendix C - Table S1. PRISMA report checklist.**  Table S1. PRISMA 2009 Checklist | | | |
| --- | --- | --- | --- |
| Section/topic | # | Checklist item | Reported on page # |
| TITLE | | |  |
| Title | 1 | Identify the report as a systematic review, meta-analysis, or both. | Page 1 |
| Structured summary | 2 | Provide a structured summary including, as applicable: background; objectives; data sources; study eligibility criteria, participants, and interventions; study appraisal and synthesis methods; results; limitations; conclusions and implications of key findings; systematic review registration number. | Page 2,3 |
| INTRODUCTION | | |  |
| Rationale | 3 | Describe the rationale for the review in the context of what is already known. | Page 4, 5 |
| Objectives | 4 | Provide an explicit statement of questions being addressed with reference to participants, interventions, comparisons, outcomes, and study design (PICOS). | Page 6 |
| METHODS | | |  |
| Protocol and registration | 5 | Indicate if a review protocol exists, if and where it can be accessed (e.g., Web address), and, if available, provide registration information including registration number. | NA |
| Eligibility criteria | 6 | Specify study characteristics (e.g., PICOS, length of follow-up) and report characteristics (e.g., years considered, language, publication status) used as criteria for eligibility, giving rationale. | Page 6, 7 |
| Information sources | 7 | Describe all information sources (e.g., databases with dates of coverage, contact with study authors to identify additional studies) in the search and date last searched. | Page 6 |
| Search | 8 | Present full electronic search strategy for at least one database, including any limits used, such that it could be repeated. | Page 6, 7 |
| Study selection | 9 | State the process for selecting studies (i.e., screening, eligibility, included in systematic review, and, if applicable, included in the meta-analysis). | Page 6, 7 |
| Data collection process | 10 | Describe method of data extraction from reports (e.g., piloted forms, independently, in duplicate) and any processes for obtaining and confirming data from investigators. | Page 7, 8 |
| Data items | 11 | List and define all variables for which data were sought (e.g., PICOS, funding sources) and any assumptions and simplifications made. | Page 8,9 |
| Risk of bias in individual studies | 12 | Describe methods used for assessing risk of bias of individual studies (including specification of whether this was done at the study or outcome level), and how this information is to be used in any data synthesis. | Page 8,9 |
| Summary measures | 13 | State the principal summary measures (e.g., risk ratio, difference in means). | Page 8,9 |
| Synthesis of results | 14 | Describe the methods of handling data and combining results of studies, if done, including measures of consistency (e.g., I2) for each meta-analysis. | Page 8,9 |
| Risk of bias across studies | 15 | Specify any assessment of risk of bias that may affect the cumulative evidence (e.g., publication bias, selective reporting within studies). | Page 8,9 |
| Additional analyses | 16 | Describe methods of additional analyses (e.g., sensitivity or subgroup analyses, meta-regression), if done, indicating which were pre-specified. | Page 8,9 |
| RESULTS | | |  |
| Study selection | 17 | Give numbers of studies screened, assessed for eligibility, and included in the review, with reasons for exclusions at each stage, ideally with a flow diagram. | Page 9-11 |
| Study characteristics | 18 | For each study, present characteristics for which data were extracted (e.g., study size, PICOS, follow-up period) and provide the citations. | Page 9-11 |
| Risk of bias within studies | 19 | Present data on risk of bias of each study and, if available, any outcome level assessment (see item 12). | Page 13,14 |
| Results of individual studies | 20 | For all outcomes considered (benefits or harms), present, for each study: (a) simple summary data for each intervention group (b) effect estimates and confidence intervals, ideally with a forest plot. | Page 9,10 |
| Synthesis of results | 21 | Present results of each meta-analysis done, including confidence intervals and measures of consistency. | Pages 4-6 |
| Risk of bias across studies | 22 | Present results of any assessment of risk of bias across studies (see Item 15). | Page 11-13 |
| Additional analysis | 23 | Give results of additional analyses, if done (e.g., sensitivity or subgroup analyses, meta-regression [see Item 16]). | Page 13,14 |
| DISCUSSION | | |  |
| Summary of evidence | 24 | Summarize the main findings including the strength of evidence for each main outcome; consider their relevance to key groups (e.g., healthcare providers, users, and policy makers). | Page 14-17 |
| Limitations | 25 | Discuss limitations at study and outcome level (e.g., risk of bias), and at review-level (e.g., incomplete retrieval of identified research, reporting bias). | Page 17,18 |
| Conclusions | 26 | Provide a healthy interpretation of the results in the context of other evidence, and implications for future research. | Page 18 |
| FUNDING | | |  |
| Funding | 27 | Describe sources of funding for the systematic review and other support (e.g., supply of data); role of funders for the systematic review. | Page 18 |

*From:* Moher D, Liberati A, Tetzlaff J, Altman DG, The PRISMA Group (2009). Preferred Reporting Items for Systematic Reviews and Meta-Analyses: The PRISMA Statement. PLoS Med 6(7): e1000097. doi:10.1371/journal.pmed1000097

**Appendix D - Table S2. Quality assessment of included studies.**

| Table S2. Quality assessment of included studies | | | | |
| --- | --- | --- | --- | --- |
| Reference | Quality assessment (NOS) | | | Total score |
|  | Selection | Comparability | Outcome |  |
| Pan L, et al. (2018) | 3 | 2 | 3 | 8 |
| Shutt RH, et al. (2017). | 3 | 2 | 2 | 7 |
| Lim YH, et al. (2017). | 3 | 2 | 3 | 8 |
| Chen SY, et al. (2017). | 3 | 1 | 2 | 6 |
| Lee MS, et al. (2016). | 3 | 2 | 2 | 7 |
| Xie Y et al. (2016). | 3 | 2 | 3 | 8 |
| Peters A, et al. (2015). | 3 | 2 | 3 | 8 |
| Liu WT, et al. (2015). | 3 | 2 | 3 | 8 |
| Lee MS, et al. (2014). | 3 | 2 | 2 | 7 |
| Xu MM, et al. (2013). | 3 | 2 | 3 | 8 |
| Bartell SM, et al. (2013). | 3 | 2 | 3 | 8 |
| Shields KN, et al. (2013). | 3 | 2 | 2 | 7 |
| Huang J, et al. (2013). | 3 | 2 | 3 | 8 |
| Jia X, et al. (2012). | 3 | 2 | 2 | 7 |
| Rich DQ, et al. (2012). | 3 | 2 | 2 | 7 |
| Hampel R, et al. (2012). | 3 | 2 | 3 | 8 |
| Huang W, et al. (2012). | 3 | 2 | 3 | 8 |
| Wu CF, et al. (2010). | 3 | 2 | 2 | 7 |
| Wu S, et al. (2010). | 3 | 2 | 2 | 7 |
| Chuang KJ, et al. (2007). | 3 | 2 | 2 | 7 |
| Zanobetti A, et al. (2010). | 3 | 1 | 3 | 7 |
| Suh HH, et al. (2010). | 3 | 2 | 3 | 8 |
| Schneider A, et al. (2010). | 3 | 2 | 3 | 8 |
| Folino AF, et al. (2009). | 3 | 2 | 3 | 8 |
| Yeatts K, et al. (2007). | 3 | 2 | 3 | 8 |
| Adar SD, et al. (2007). | 3 | 2 | 2 | 7 |
| Luttmann-Gibson H, et al. (2006). | 3 | 2 | 3 | 8 |
| Wheeler, A, et al. (2006). | 3 | 2 | 3 | 8 |
| Timonen KL, et al. (2006). | 3 | 2 | 3 | 8 |
| Riediker M, et al. (2004). | 3 | 2 | 2 | 7 |
| Schwartz J, et al. (2005). | 3 | 2 | 2 | 7 |
| Magari SR, et al. (2002). | 3 | 2 | 2 | 7 |
| Brauer M, et al. (2001). | 3 | 2 | 3 | 8 |

| **Appendix- Table S3. Publication bias** | | | |
| --- | --- | --- | --- |
| Table S3. Publication bias. | | | |
|  | | | |
| HRV indices | No. of studies | Begg’s test | Egger’s tests |
| SDNN | 29 | 0.822 | 0.007 |
| rMSSD | 27 | 0.260 | 0.315 |
| HF | 24 | 0.766 | 0.305 |
| LF | 16 | 0.602 | 0.180 |
| HRV, heart rate variability; SDNN, the standard deviation of NN intervals; rMSSD, the square root of the mean of the squares of the successive differences between adjacent NNs; HF, frequency power; LF, low frequency power. | | | |

| **Appendix F - Table S4: Meta-regression analysis by potential modifier** | | | | | | | |
| --- | --- | --- | --- | --- | --- | --- | --- |
| Table S4: Meta-regression analysis by potential modifier | | | | | | | |
| **Subgroup** | **Grouping criteria** | **No. of studies** | **Meta-regression** | | | | |
|  |  |  | **Coef.** | ***P* value** | | **I^2^** | |
| **Study location** | | | | | | | |
| SDNN | Asian | 10 | Ref. | | 0.851 | | 83.79% |
|  | European | 4 | 0.55 (-1.76, 2.87) | |  |  |  |
|  | North American | 15 | 0.37 (-1.30, 2.04) | |  |  |  |
| rMSSD | Asian | 9 |  | | 0.716 | | 76.32% |
|  | European | 5 | 0.80 (-1.35, 2.95) | |  |  |  |
|  | North American | 13 | 0.05 (-1.79, 1.89) | |  |  |  |
| HF | Asian | 12 | Ref. | | 0.622 | | 87.99% |
|  | European | 2 | -2.53 (-8.51, 3.44) | |  |  |  |
|  | North American | 10 | -1.17 (-4.58, 2.23) | |  |  |  |
| LF | Asian | 10 | Ref. | | 0.861 | | 73.68% |
|  | European | 0 | - | |  |  |  |
|  | North American | 6 | 0.27 (-3.04, 3.59) | |  |  |  |
| **PM_2.5_ assessment** | | | | | | | |
| SDNN | Fixed site or others | 15 | Ref. | | 0.867 | | 85.19% |
|  | Individual monitor | 14 | 0.12 (-1.39, 1.63) | |  |  |  |
| rMSSD | Fixed site or others | 17 | Ref. | | 0.917 | | 77.31% |
|  | Individual monitor | 10 | 0.08 (-1.55, 1.71) | |  |  |  |
| HF | Fixed site or others | 13 | Ref. | | 0.060 | | 89.08% |
|  | Individual monitor | 11 | -2.77 (-5.67, 0.13) | |  |  |  |
| LF | Fixed site or others | 10 | Ref. | | 0.682 | | 72.72% |
|  | Individual monitor | 6 | 0.61 (-2.52, 3.75) | |  |  |  |
| **Health status** | | | | | | | |
| SDNN | Patients | 13 | Ref. | | 0.927 | | 84.38% |
|  | Healthy population | 16 | 0.07 (-1.44, 1.58) | |  |  |  |
| rMSSD | Patients | 17 | Ref. | | 0.041 | | 66.02% |
|  | Healthy population | 10 | -1.58 (-3.08, -0.07) | |  |  |  |
| HF | Patients | 13 | Ref. | | 0.082 | | 89.14% |
|  | Healthy population | 11 | -2.62 (-5.60, 0.36) | |  |  |  |
| LF | Patients | 8 | Ref. | | 0.537 | | 74.18% |
|  | Healthy population | 8 | -0.91 (-4.01, 2.18) | |  |  |  |
| **Age** | | | | | | | |
| SDNN^*^ |  | 28 | 0.009 (-0.032, 0.050) | | 0.650 | | 85.64% |
| rMSSD |  | 27 | -0.015 (-0.051, 0.021) | | 0.391 | | 79.72% |
| HF |  | 24 | -0.054 (-0.130, 0.022) | | 0.156 | | 84.96% |
| LF |  | 16 | -0.037 (-0.109, 0.035) | | 0.288 | | 69.73% |
| **PM_2.5_ levels** | | | | | | | |
| SDNN |  | 29 | 0.011 (-0.017, 0.038) | | 0.447 | | 85.34% |
| rMSSD^*^ |  | 26 | 0.010 (-0.020, 0.041) | | 0.495 | | 80.33% |
| HF |  | 24 | -0.002 (-0.051, 0.047) | | 0.925 | | 89.14% |
| LF |  | 16 | 0.009 (-0.049, 0.068) | | 0.735 | | 73.35% |
| Ref. reference; HRV, heart rate variability; SDNN, standard deviation of all normal-to-normal intervals; rMSSD, root mean square of | | | | | | | |
| successive differences in adjacent normal-to-normal intervals; HF, high frequency power; LF, low frequency power; * there were 1 study included in meta-regression did not report age or PM_2.5_ levels; Mean or median of age and PM_2.5_ levels were used in meta-regression. If a study only reported the age range of participants, the average of the maximum and minimum age was used. If a study only reported the minimum age of participants, the minimum age was used in meta-regression. | | | | | | | |

**Appendix G - Figure S1. Meta-regression plots of percent change of HRV and age of participants.**

| 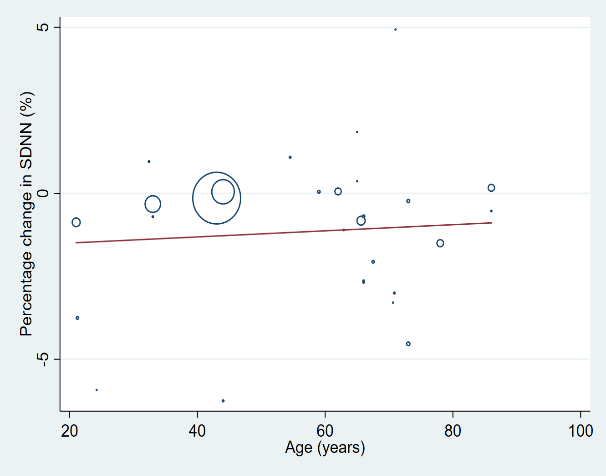 | 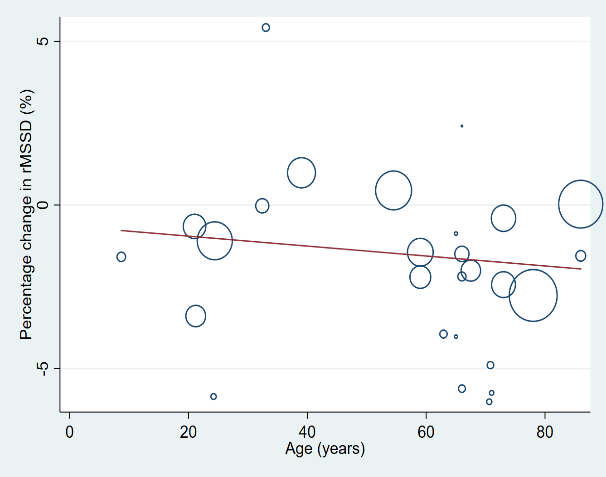 |
| --- | --- |
| 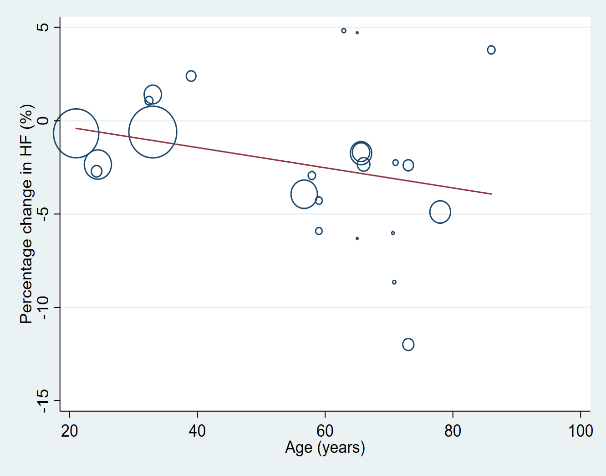 | 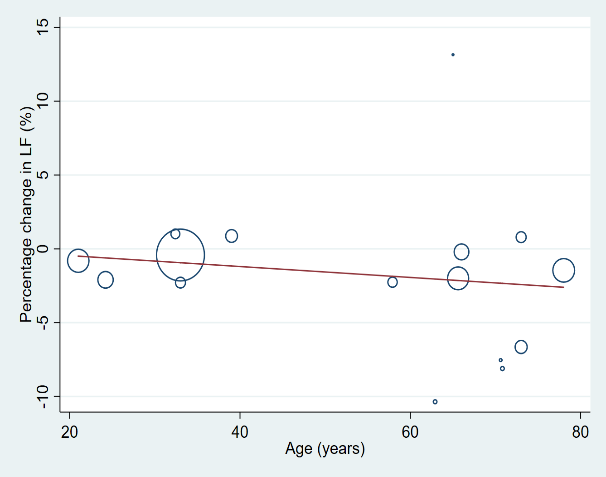 |

**Figure S1**. Meta-regression plots of percent change of HRV and age of participants.

**Appendix H- Figure S2. Meta-regression plots of percent change of HRV and PM_2.5_ levels.**

| 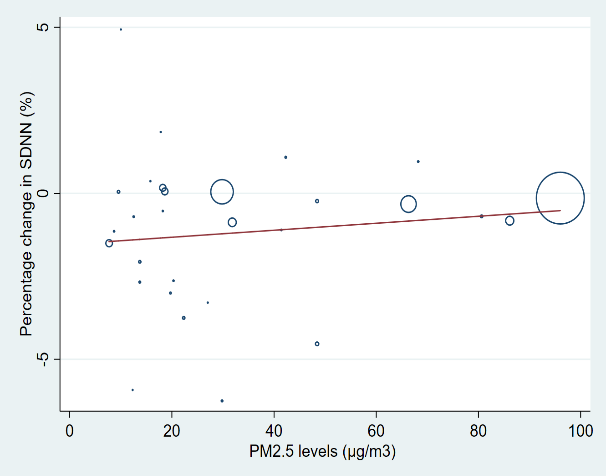 | 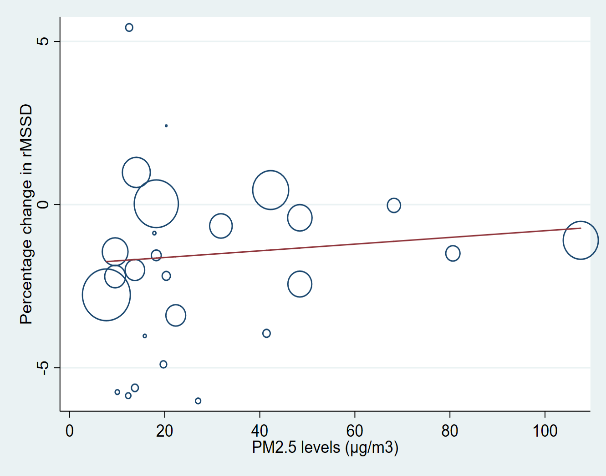 |
| --- | --- |
| 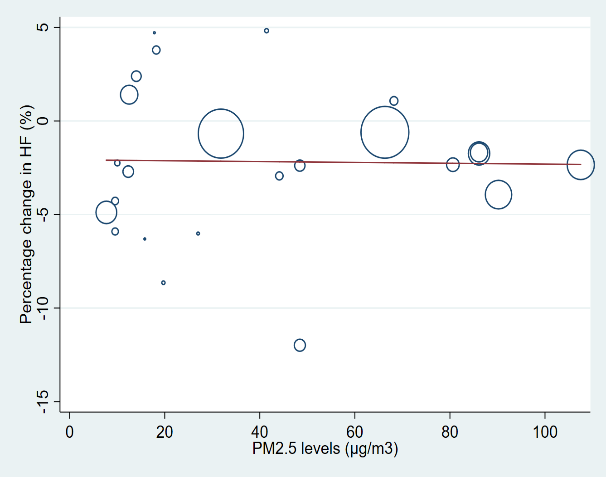 | 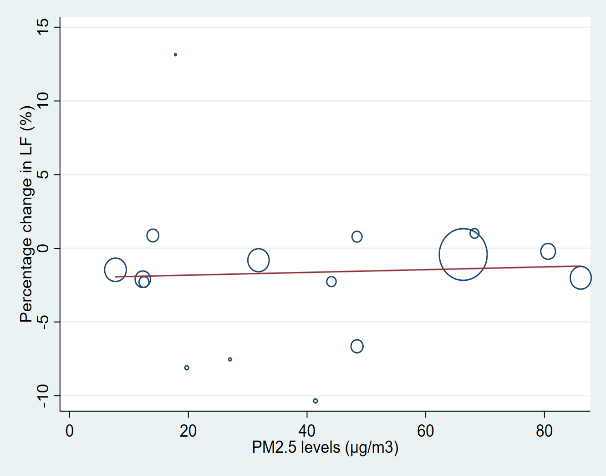 |

**Figure S2**. Meta-regression plots of percent change of HRV and PM_2.5_ levels.

**Appendix H - Figure S3. Funnel plots of publication bias analyses.**


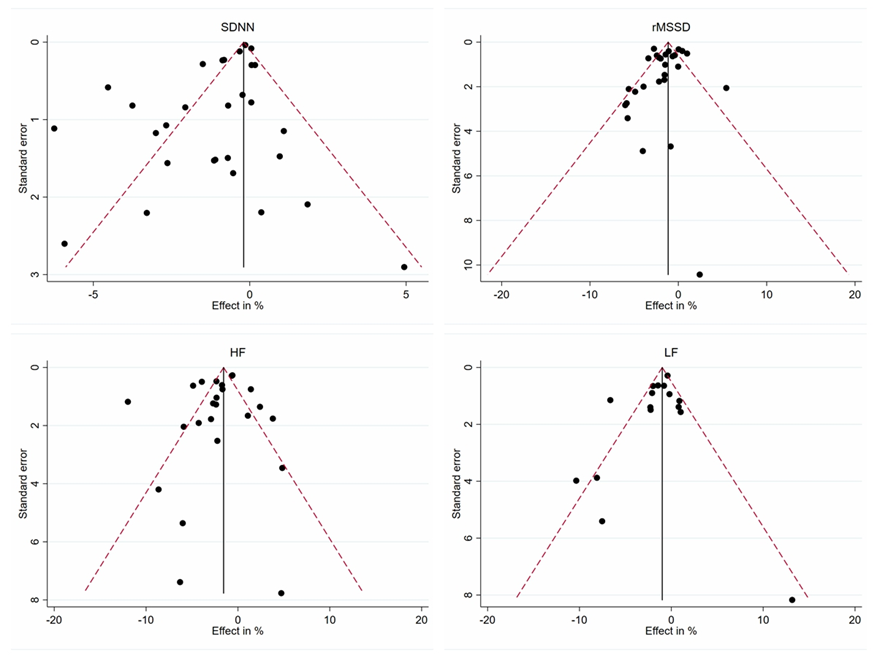


**Figure S3.** Funnel plots of publication bias analyses.

**Appendix I- Figure S4. Sensitivity analysis the lower and upper limits of the pooled results after omitting one study each at a time.**

| SDNN 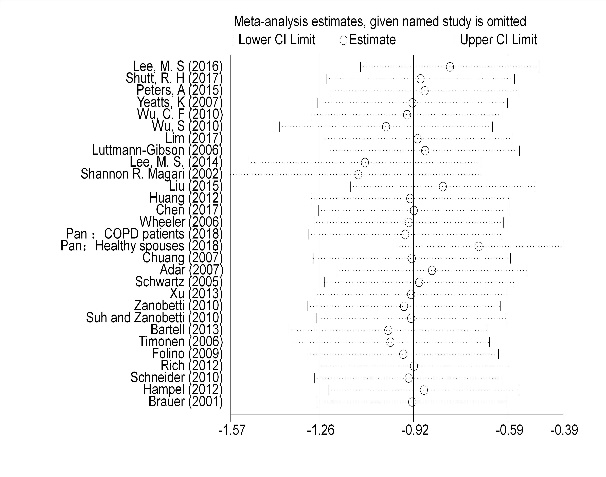 | rMSSD 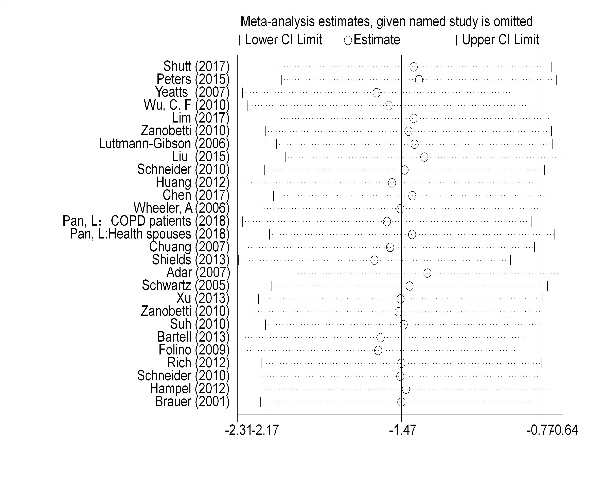 |
| --- | --- |
| HF 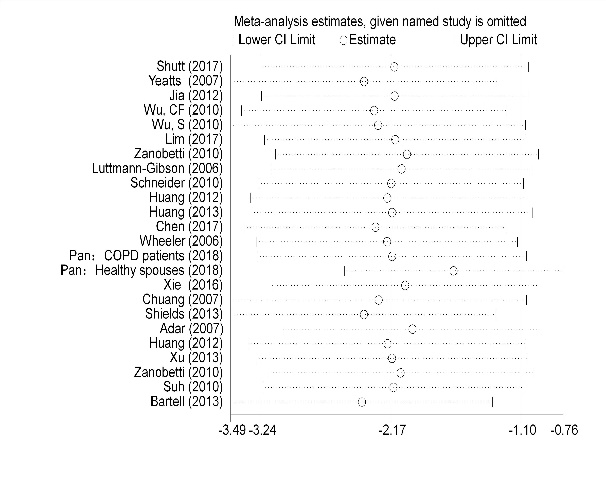 | LF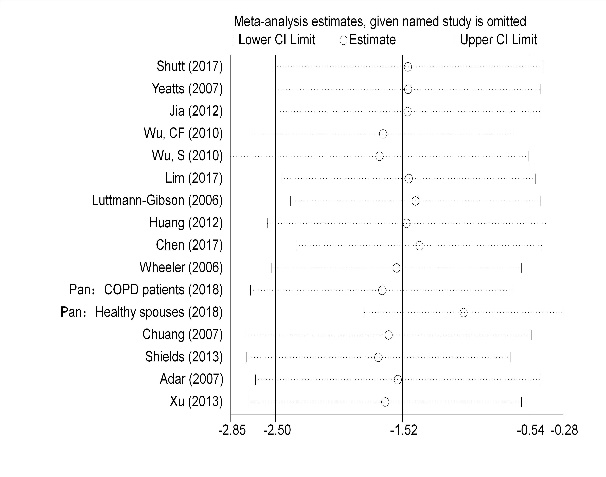 |

**Figure S4.** Sensitivity analysis the lower and upper limits of the pooled results after omitting one study each at a time.
